# Hereditary predisposition of water voles (Arvicola amphibius L.) to seizures in response to handling

**DOI:** 10.18699/VJGB-22-45

**Published:** 2022-07

**Authors:** G.G. Nazarova, L.P. Proskurnyak

**Affiliations:** Institute of Systematics and Ecology of Animals of the Siberian Branch of the Russian Academy of Sciences, Novosibirsk, Russia; Institute of Systematics and Ecology of Animals of the Siberian Branch of the Russian Academy of Sciences, Novosibirsk, Russia

**Keywords:** water vole, seizures, age, hereditary predisposition, population cycle, life span, водяная полевка, судорожные припадки, возраст, наследственная предрасположенность, циклы численности, продолжительность жизни

## Abstract

Finding out the hereditary predisposition to seizures in response to specif ic external stimuli is important for understanding the causes of epileptiform conditions, developing new methods for their prevention and therapies. In the water vole, individuals with convulsive seizures are found both in natural and laboratory conditions. The data of long-lasting maintenance and breeding of water voles in vivarium conditions were analyzed in order to establish a hereditary predisposition to convulsive seizures, and the inf luence of sex and age on their development. In the vivarium, seizures are provoked by handling and are observed in 2.4 % of voles caught in the natural population with cyclic f luctuations in abundance. Seizures are observed more often in individuals caught in the phases of decline and depression of abundance than in individuals caught in the phases of rise or peak. Convulsive states are probably an element of adaptive behavior formed in the predator-prey system. In natural conditions, individuals predisposed to convulsive seizures may have a selective advantage when under increasing pressure from predators. Convulsive seizures in response to handling were noted in 29.8 % of descendants of captive-bred water voles. The proportion of such individuals increased signif icantly if one or both parents had convulsive states, which indicates the presence of a hereditary predisposition to seizures. In parent–offspring pairs, a signif icant correlation was found between the average age of onset of the f irst seizures in parents and their offspring, r = 0.42, p <0.01. The minimum age of registration of seizures in the water vole is 39 days, the maximum is 1105 days, and the median is 274 days. Predisposition to seizures is not related to sex. Genes that control the occurrence of seizures have a pleiotropic effect on life span, since individuals with seizures live longer in vivarium conditions than individuals with a normal phenotype. The water vole can serve as a suitable model object for studying the nature of convulsive states and the evolution of longevity

## Abstract (ru)

Выяснение наследственной предрасположенности к судорогам в ответ на специфические внешние стимулы важно для понимания причин эпилептиформных состояний, нахождения новых методов их предупреждения и лечения. Особи водяной полевки с судорожными припадками встречаются как в природных, так и лабораторных условиях. Проанализированы данные многолетнего содержания и разведения водяных полевок в условиях вивария с целью установления наследственной предрасположенности к судорожным припадкам и влияния пола и возраста на их развитие. В условиях вивария припадки были спровоцированы взятием в руки и отмечены у 2.4 % полевок, отловленных в природной популяции с циклическими колебаниями численности. Судорожные припадки чаще встречали у особей, отловленных в фазы спада и депрессии численности, чем у особей с фаз подъема или пика. Судорожные состояния, вероятно, служат элементом адаптивного поведения, сформировавшегося в системе «хищник–жертва». В природных условиях особи, предрасположенные к судорожным припадкам, могут иметь селективное преимущество при усилении пресса хищников. Судорожные припадки в ответ на хэндлинг отмечены у 29.8 % потомков водяных полевок виварного разведения. Доля таких особей существенно увеличивалась, если у одного или обоих родителей регистрировали судорожные состояния, что свидетельствует о наследственной предрасположенности к припадкам. В парах «родители–потомки» обнаружена достоверная корреляция среднего возраста наступления первых припадков (r = 0.42, p < 0.01). Минимальный возраст регистрации припадков у водяной полевки составляет 39 дней, максимальный – 1105, медианный – 274 дня. Предрасположенность к припадкам не связана с полом. Гены, контролирующие возникновение судорожных состояний, оказывают плейотропное действие на продолжительность жизни: особи с судорожными припадками в условиях вивария живут дольше, чем особи с нормальным фенотипом. Водяная полевка может служить подходящим модельным объектом для изучения природы судорожных состояний и эволюции долголетия.

## Introduction

Tonic-clonic seizures – uncontrolled muscle tension or contraction – can occur in humans and other mammals: gray rats Rattus norvegicus (Poletaeva et al., 2017), house mice Mus musculus (Skradski et al., 1998), Mongolian gerbils Meriones unguiculatus (Buchhalter, 1993), deer mice Peromyscus maniculatus (Jackson, 1997), Syrian hamsters Mesocricetus auratus (Muñoz et al., 2017), meadow voles Microtus pennsylvanicus (Bronson and De La Rosa, 1994), dogs Canis familiaris (Catala et al., 2018), cats Felis catus (Pakozdy et al., 2014), and rabbits (Gülersoy et al., 2021). The most common cause of seizures is an imbalance between excitatory and inhibitory neurotransmitter mechanisms in the brain (Poletaeva et al., 2017). Seizures are categorized into spontaneous (provoked by unknown factors) and reflex ones (caused by specific external stimuli, for example, electrical, auditory, visual, or tactile) (Okudan, Özkara, 2018).

In response to tactile stimulation during handling, prolonged tonic-clonic seizures take place in bank voles Myodes glareolus (Schønecker, 2009), meadow voles M. pennsylvanicus (Bronson, De La Rosa, 1994), and Mongolian gerbils M. unguiculatus (Ludvig et al., 1991; Buckmaster, 2006). Other stimuli (e. g., audiogenic or olfactory) are not effective at inducing seizures in these species. The gray rat is a popular experimental model for research on audiogenic epilepsy (Poletaeva et al., 2017). It should be noted that genetic factors predisposing to various types of reflex epilepsy are poorly understood, and genes that control somatosensory epilepsy are not known at all (Okudan, Özkara, 2018). Clarification of hereditary predisposition to handling-induced seizures is important for understanding the causes of seizures and for improving the prevention and treatment methods.

In recent decades, rodents have been widely used in neurophysiological investigation into epilepsy owing to their small size, peacefulness, and rapid reproduction in captivity (Jackson, 1997). Comparative studies on mammals are needed to better understand the evolutionary and genetic factors that explain seizures because they should help to find common components of the pathogenesis of this neurological disorder that are of clinical relevance to humans (Grone, Baraban, 2015). To elucidate the evolutionary mechanisms underlying the onset of convulsive states, species in which individuals with seizures occur under natural conditions are of great interest. L.G. Krotova (1962) – who has investigated the function of adrenal glands and carbohydrate metabolism in water voles (Arvicola amphibius L.) living in the floodplain of the Chusova River (Sverdlovsk Oblast, Russia) – noticed that some captured animals had seizures accompanied by a coma. Owing to detailed complex research data on various characteristics of population ecology, genetics, and physiology of the water vole and to excellent advances in the methods for its breeding in captivity (The Water vole…, 2001), the water vole can become a promising experimental model for investigation into predisposing factors of seizures at populational, genetic, and neurological levels.

In this study, data are analyzed that were obtained during long-term breeding of water voles at an animal facility to achieve the following aims: 1) to describe the picture of the seizures; 2) to estimate the proportion of individuals with seizures among animals caught in the wild and among animals born in the vivarium; 3) to determine the minimal, maximal, and average age at onset of seizures; 4) to find out whether the manifestation of convulsive seizures is associated with the sex of these animals and/or the presence of this neuropathology in the parents; and 5) to identify a possible relation between the predisposition to seizures and the life span.

## Materials and methods

The study was performed on two groups of water voles kept at the animal facility of the Institute of Systematics and Ecology of Animals of the Siberian Branch of the Russian Academy of Sciences (Novosibirsk, Russia), under natural lighting conditions with free access to water and feed (carrots, well-steamed grain mixtures, and fresh greens):

1) the wild group (n = 589), i. e., animals that were caught during 1983–2017 in the Ubinsky District of Novosibirsk Oblast (Russia), where comprehensive ecological and physiological studies on a population with pronounced high-amplitude fluctuations of population size (6–7-year cycle) were being conducted (The Water vole …, 2001; Evsikov et al., 2017). After capture, these water voles lived in the vivarium for at least 3 months (290 females and 299 males). Ninety-four voles from the peak phase, 32 from the decline phase, 155 from the depression phase, and 308 from the increase phase were analyzed;

2) the vivarium group (n = 1776), i. e., water voles (parents and offspring) that were born in the vivarium and survived at least 22 days after birth. This group was founded by individuals caught in the aforementioned natural population. To preserve genetic heterogeneity, the group was regularly replenished with wild individuals: at least once every 3 years.

This approach allows, on the one hand, to identify populational factors that affect the prevalence of convulsive states in the wild, and on the other hand, to identify genetic and ontogenetic factors predisposing parents and offspring to seizures under standard maintenance conditions in a vivarium.

The work on the animals of the two groups throughout the study period was done according to a standard scheme: 1) all water voles were individually marked, sexed, and dates of birth (capture) and death were recorded; 2) until the age of 70 days, weekly, the water voles born in the vivarium were weighed and their body length was measured; the weight and body length of the vivarium animals older than 70 days and those of individuals caught in the natural population were measured once a month; 3) during the reproduction period, before forming of mating pairs (closely related animals were not used for setting up mating pairs), vaginal smears were taken from females, and anogenital distance was measured in males; 4) throughout their life span, the water voles were subjected to experiments aimed at examining the relation between ethological–physiological characteristics of water voles and their reproductive ability. The following procedures were performed: tests of social interactions, of olfactory mate choice, and of parental behavior as well as blood sampling from the retroorbital sinus and tail, urine collection. During the experiments, water voles were subjected to handling (at least once a month), and some animals developed convulsive seizures. The main diagnostic criterion of a convulsive state was assumed to be pronounced tonic or clonic contractions of muscles of the trunk and/or limbs. Such cases were recorded. The animals with convulsive seizures were not culled and were allowed to reproduce. There were no spontaneous seizures when the animals were in home cages and were not subjected to the handling.

Statistical analysis was performed in SPSS Statistics 15.0 (IBM Corp., USA) and Statistica 6.1 (StatSoft Inc., USA) software. Comparisons of proportions (%) between groups were conducted by Pearson’s c2 test. Effects of categorical variables and continuous variables on age at onset of the first seizure (hereafter: “age at seizure onset”) or on the life span were assessed via the linear mixed model, with dependent variables subjected to the natural-logarithm (ln) transformation in order to make their distributions normal. Survival curves were constructed by the Kaplan–Meier method. Differences between the curves were evaluated by the Gehan–Wilcoxon test. The text and tables show means of parameters (X ), standard error (± SE), and sample size (n). Statistical significance was assumed at p < 0.05.

## Results

Description of the seizures

Each seizure was convulsive (tonic and/or clonic) and occurred in the first minute after a water vole was subjected to handling. The seizure began with a twitching of the vibrissae and tonic tension of anterior trunk muscles, followed by their rhythmic contractions; meanwhile, the animal was likely to arch its back and tilt its head back. At the first signs of a seizure, individuals were placed into an arena, and their state was examined. The convulsions spread throughout the whole body, and in some cases, the seizure was accompanied by gnashing of teeth and very rarely by loss of consciousness. After the seizures stopped, the animals experienced locomotor agitation: “wild running”.

The proportion of individuals with convulsive seizures among water voles taken from the natural population during different phases of cyclic population dynamics

Seizures were noted in 2.4 % of the 589 water voles in the wild group; the seizures were caused by handling. There were no sex differences in susceptibility to seizures (χ2 = 0.004, p = 0.950).

**Fig. 1.**
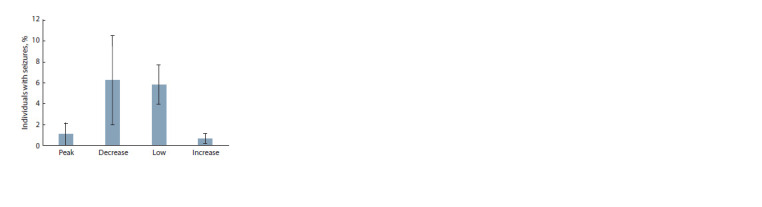
The proportion of individuals (%) with seizures in different phases of population cycle

The proportion of individuals with seizures was affected by the population phase at which the animals were taken from the population (χ2 = 14.58, df = 3, p = 0.002), Fig. 1. Among the water voles from the decline or low phase, the proportion of those susceptible to seizures was significantly higher than that among the water voles from the increase or peak phase (5.88 ± 1.72 % and 0.97 ± 0.56 %, respectively; χ2 = 10.25, df = 1, p = 0.014).

Seizures among the water voles born in the vivarium

Seizures were observed in 29.8 % of the 1776 animals in the vivarium group. Susceptibility to seizures was not influenced by sex (males: 29.4 %, females: 30.1 %; χ2 = 0.116, df = 1, p = 0.734). There were no seizures during the suckling period, which ended at age 3 weeks.

Age at seizure onset

**Fig. 2.**
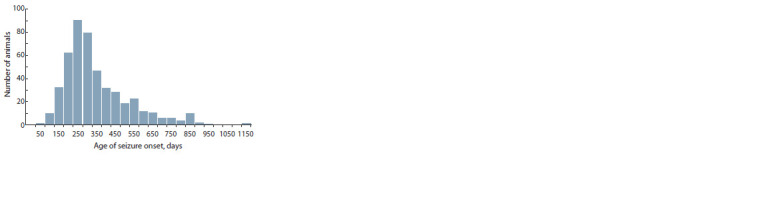
The histogram of the distribution of age of seizure onset.

The date of onset of the first seizure was recorded only for 480 animals. The minimum age at seizure onset was 39 days, and the maximum age at seizure onset was 1105 days. In the seizure group, modal age was 189 days, median age 274 days, and the mean age 324.6 ± 7.8 days. A histogram of the distribution of age at seizure onset is presented in Fig. 2.

Analysis of the data using a mixed linear model, taking into account the year of birth and litter identity (ID) as random factors, showed no effect of sex as a fixed factor (F1,416.5 = = 0.312, p = 0.577) on the age of onset of the first seizure (males: 351.5 ± 25.3 days, females: 344.4 ± 25.1 days). The influence of the year of birth and lD was significant (Z = 2.877, p = 0.004 and Z = 3.841, p < 0.001, respectively).

Hereditary predisposition to convulsive states

To clarify hereditary predisposition of water voles to handlinginduced seizures, we used animals that lived in the vivarium for at least 39 days, which was the minimum age at seizure onset in this work. Thus, 1656 water voles from 445 litters were analyzed. The results indicated that the susceptibility of offspring to seizures correlates with seizure status of their parents (Table 1). Namely, the proportion of offsprings with seizures was significantly higher if one or both parents had seizures (χ2 = 151.67, df = 3, p < 0.001).

**Table 1.**
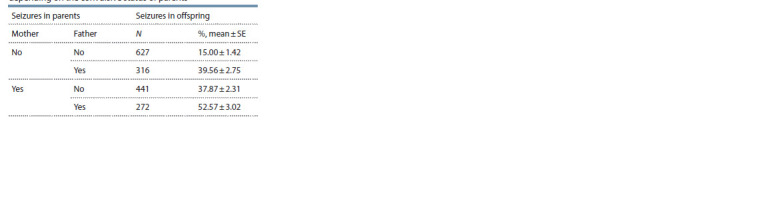
The percentage of offspring with seizures depending on the convulsive status of parents

**Fig. 3.**
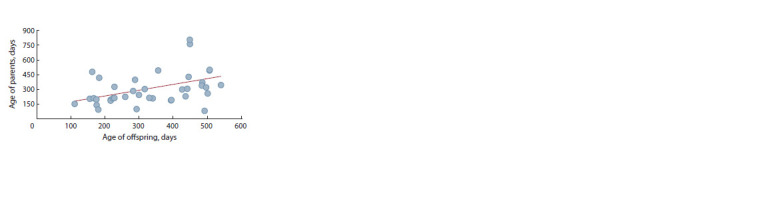
The correlation between mean ages of seizure onsets within “parent–offspring” pairs.

Within “parent–offspring” pairs with known age at seizure onset in both parents and offspring (39 parental pares, 90 offspring), a significant correlation between average ages at seizure onset was found (r = 0.42, p < 0.01; Fig. 3), indicating a significant additive contribution of genes to this trait and the possibility of selection for “age at seizure onset”.

The relation between the presence of seizures and the life span

**Fig. 4.**
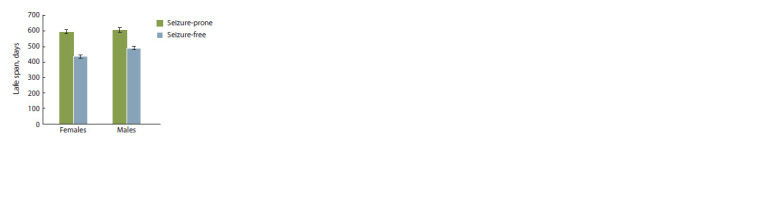
The relation between the life span and the predisposition to seizures among male and female water voles.

To clear up the relation between the presence of seizures and the life span, the linear mixed model was used, which included categorical variables (sex and the presence/absence of seizures) and the birth year and ID as random factors. As a result, we detected significant effects of sex (F1,1410.3 = 5.342, p = 0.021) and of seizure status of an individual (F1,1392.2 = = 132.926, p < 0.001) on the life span. Males lived longer than females (β = 24.041 ± 10.402, t = 2.311, p = 0.021). The life span of animals without seizures was significantly shorter than that of animals with seizures (β = –134.458 ± 10.402, t = –11.529, p <0.001), Fig. 4. A significant impact of the birth year on the life span was noticed (Wald-Z = 3.295, p = 0.001), whereas such an effect of ID was statistically insignificant (Wald-Z = 1.419, p = 0.156).

**Fig. 5.**
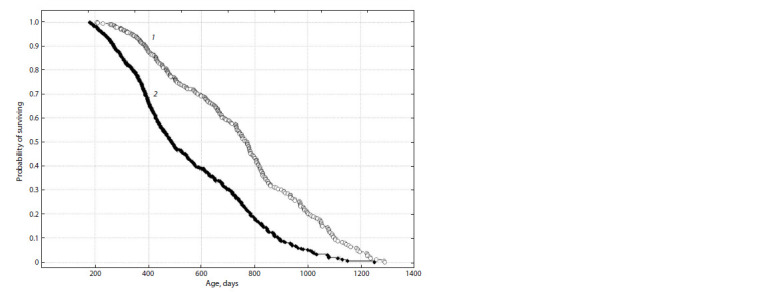
Survival curves of two groups of water voles: seizure-prone (1) and seizure-free (2).

Figure 5 shows survival curves of two groups of water voles (with seizures and without seizures). Significant differences were found between them according to the Gehan–Wilcoxon test (GW = –11.452, p < 0.001).

The effect of seizure status of parents on the life span of the offspring

To clarify the dependence of life span on the convulsive status of parents, using a linear mixed model, we assessed the influence of fixed factors: sex, predisposition to convulsions of the mother, father and interaction of parents’ phenotypes on the life span of offspring. The birth year and ID were included in the model as random factors. It was found that the life span is affected by sex (F1,1413.6 = 4.580, p = 0.033), by maternal seizure status (F1,366.2 = 7.280, p = 0.007), and by an interaction of these maternal and paternal phenotypes (F1,376.4 = = 4.024, p = 0.046). The offspring of parental pairs in which either the mother or both parents did not have seizures manifested a significantly shorter life span (β = –39.442 ± 12.664, t = –3.115, p = 0.002 and β = –50.504 ± 25.176, t = –2.006, p = 0.046, respectively). The effect of paternal seizure status on the offspring lifespan was not statistically significant (F1,393.5 = 0.253, p = 0.615), and neither was the effect of ID (Wald-Z = 1.760, p = 0.078). Data on the mean life span of offspring and seizure status of the parents is given in Table 2.

**Table 2.**
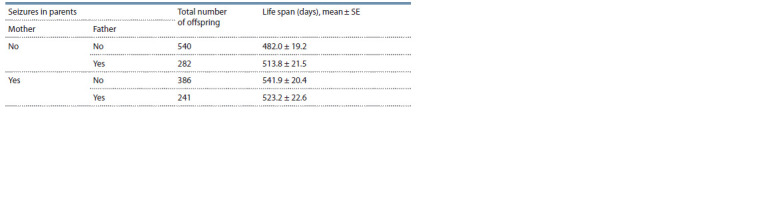
Mean life span of the offspring in relation to seizure status of the parents

## Discussion

These studies establish the occurrence of genetically based, tonic-clonic convulsions in water voles in response to handling. The incidence of seizures did not depend on sex. Prolonged tonic-clonic seizures in response to handling have been documented for the bank vole (Schønecker, 2009), the meadow vole (Bronson, De La Rosa, 1994), and the Mongolian gerbil (Ludvig et al., 1991; Buckmaster, 2006). The pattern of seizures in the water vole turned out to be similar to that in bank voles, meadow voles, and Krushinsky–Molodkina audiogenic rat strain, except that in bank and meadow voles, there is no “wild running” phase observed in Krushinsky– Molodkina rats (Bronson, De La Rosa, 1994; Schønecker, 2009; Poletaeva et al., 2017) and in water voles.

According to the literature, reflex epileptiform seizures caused by tactile or acoustic stimuli are typical only for small rodents, which is associated with the structural and functional features of their central nervous system, due to which pathological, from a human point of view, behavioral responses to stimuli that inform about danger are carried out (Poletaeva et al., 2017; Fedotova et al., 2021). Convulsive states are probably a component of adaptive behavior formed in a “predator– prey” system. Short-term clonic convulsions in response to tactile or acoustic stimulation can have a frightening effect on raptors that capture prey with their claws, and rapid running, as the next phase of a convulsive seizure, allows the prey to quickly take refuge in a safe place.

Individuals susceptible to seizures may have a selective advantage during high predator pressure. It is known that the number of specialized bird predators with great mobility changes synchronously with the dynamics of population size of water voles (Weber et al., 2002). The results of crossings of water voles, differing in their predisposition to seizures, indicate the hereditary transmission of this trait: the proportion of offspring with seizures significantly increases if one or, especially, both parents had seizures. Therefore, an increase in the prevalence of individuals with seizures during the phases of decline and depression of cyclic population dynamics may be explained by positive selection for seizure susceptibility at the peak phase of population numbers accompanied by an increase in predator pressure, or greater inbreeding at the low phase, or random processes.

In our study, the minimum age at seizure onset among water voles was 39 days, the maximum age at seizure onset was 1105 days, and the median age at seizure onset, 274 days. Among bank voles, median age at seizure onset is 157 days (Schønecker, 2009). Seizures develop at a later age in voles and gerbils than in seizure-prone laboratory rats and mice.

According to our findings, in the water vole, age at seizure onset is an inherited trait that can be selected for. Identification of the main mutation that triggers seizures and elucidation of the mechanisms underlying the observed correlations between age and seizures may provide insights into the pathogenesis of seizures in other mammals. It is now known that 70–80 % of epilepsy cases are associated with one or more genetic factors, with the remaining cases attributable to acquired conditions such as a tumor, stroke, or head injury (Myers, Mefford, 2015).

Genes that control the predisposition to convulsive states in response to handling can be stored in populations due to their positive pleiotropic influence on life span. Here, we demonstrated that under vivarium conditions, individuals with seizures live longer than individuals with a normal phenotype. These results contradict mouse studies that have addressed the effects of individual genes on epileptogenesis and life span (Marshall et al., 2021); this discrepancy points to diversity of genetic mechanisms mediating the association of such a behavioral phenotype with viability.

The presence of common links in the effects of genes that control the onset of handling-induced seizures and life span confirms the positive effect of the mother’s convulsive status on the life span of the offspring in water voles. The offspring of mothers with seizures were found to live longer than the offspring of mothers with the normal phenotype, whereas the offspring life span was not affected by seizure status of the father. It is possible that genetic predisposition of females to seizures correlates with physiological parameters during pregnancy, with lactation capacity, or with maternal behavior, which in turn determine offspring viability. We are planning on investigating this topic in the future.

Thus, the water vole can serve as a suitable experimental model for researching not only the nature of convulsive states but also the evolution of longevity. Further studies are needed regarding physiological and genetic mechanisms behind the handling-induced seizures in the water vole as well as regarding the relation of seizure susceptibility to life history traits.

## Conclusion

It is shown for the first time that the water vole can have convulsive seizures in response to handling. When water voles are kept in a vivarium, the proportion of animals susceptible to convulsive seizures is higher among water voles caught in the decline or depression phase of a cycling natural population than among water voles caught in the increase or peak phase. The susceptibility to seizures is an inherited trait correlating with the life span; this finding implies the possibility of natural and artificial selection for this trait. Our data open up opportunities for the creation of water vole strains that differ in their susceptibility to handling-induced epileptiform seizures, for research on the mechanisms of epileptogenesis and longevity.

## Conflict of interest

The authors declare no conflict of interest.
